# Rural-urban disparities in Emergency Medical Services: a qualitative study of barriers and opportunities in Rivers State, Nigeria

**DOI:** 10.1016/j.afjem.2026.100946

**Published:** 2026-01-28

**Authors:** Adaeze Oreh, Folake Owodunni, Oluwaseun Adebayo Adewunmi, Ihuoma Opelia-Ezeh, Olufemi Onasanya, Sylvanus Ojum, Dede Siyeofori, Kinikanwo Green

**Affiliations:** a Rivers State Ministry of Health, Nigeria; b Emergency Response Africa (ERA), Nigeria

**Keywords:** Emergency medical services, Rural-urban discrepancies, Levesque framework

## Abstract

**Introduction:**

Emergency Medical Services (EMS) are critical for reducing morbidity and mortality in low- and middle-income countries (LMICs), yet significant disparities persist between urban and rural areas. This qualitative study explores barriers and opportunities in EMS utilisation among residents of Rivers State, Nigeria.

**Methods:**

Using a hybrid deductive-inductive approach guided by the Levesque framework for healthcare access, we conducted a focus group discussion with 20 purposively selected stakeholders representing nine categories, including healthcare providers, policymakers, community leaders and EMS users. Data was analysed thematically to identify barriers and opportunities in EMS utilisation.

**Results:**

Analysis revealed systemic barriers across five dimensions: accessibility (delayed response times, poor communication), acceptability (cultural beliefs favouring faith-based interventions), availability (inadequate infrastructure and staffing), affordability (high out-of-pocket costs), and appropriateness (gender and mental health disparities). These challenges were more pronounced in rural areas where they faced more compounded barriers, including geographic isolation and limited ambulance coverage. Several opportunities for improvement emerged, including community first-responder training, technology integration (e.g., GPS tracking), and multi-level collaborations, both inter-agency and with local non-governmental organisations (NGOs).

**Conclusion:**

The findings underscore the urgent need for policy reforms to decentralise EMS management, enhance public awareness, and address socioeconomic inequities. This study contributes to the discourse on healthcare disparities in LMICs and provides actionable recommendations for stakeholders to strengthen EMS systems in Nigeria through multi-sectoral collaboration.

**African Relevance**

• The study explores EMS disparities in Rivers State, Nigeria, addressing a public health issue prevalent across sub-Saharan Africa.

• Findings provide evidence-based, context-specific solutions that can guide regional EMS reforms and policy development.

• The work contributes to Africa’s broader health systems strengthening agenda under the UHC 2030 and Africa CDC emergency preparedness frameworks.

Alt-text: Unlabelled box dummy alt text

## Background

A medical emergency is an illness or acute injury that presents an immediate threat to life or long-term health and is usually time-sensitive [1,2], ranging from allergic reactions to cardiovascular emergencies and obstetric emergencies [3,4]. Emergencies contribute significantly to the global burden of diseases, with 41.5 % of morbidities and 50.7 % of mortalities attributed to emergency medical conditions [1]. This has led to an estimated 84 % of Disability Adjusted Life Years (DALYs) being linked to medical emergencies [5]. In health care, DALYs are years of healthy life that are lost due to injury and disease. For Nigeria, medical emergency cases account for 345 DALYs per 1000 [6,7].

Emergency Medical Services (EMS) represents the pre-hospital component of emergency care, a specialised system coordinating health personnel, equipment, and facilities to deliver prompt care at incident scenes before hospital arrival [8,9]. Throughout this manuscript, 'EMS' denotes pre-hospital emergency response systems, while 'emergency care' refers to the broader continuum including hospital-based services. This pre-hospital phase directly impacts outcomes across diverse emergencies, including trauma, cardiovascular events, and obstetric complications [10].

While high-income countries have established functional EMS systems, low- and middle-income countries (LMICs) face significant challenges, bearing disproportionate burdens of injuries and acute illnesses [11,12]. LMICs often prioritise hospital care focused on infectious diseases and maternal-child health over EMS development [13,14].

Nigeria's nascent EMS system is provided by governmental and private organisations [15]. Furthermore, Nigeria’s health system has struggled to meet the health needs of its citizens despite its economic advances [15,16] and was ranked 142 out of 195 countries when its performance on the healthcare access and quality (HAQ) index was evaluated [17].

National initiatives such as the National Emergency Medical Services and Ambulance Scheme (NEMSAS) from the 2014 National Health Act (NHA) [18] were instituted by the government to produce pre-hospital care; however, its reach remains limited in rural areas. Additional programs like the Nigeria Health Sector Renewal Investment Initiative (NHSRII) [19] and Rural Emergency Services and Maternal Transportation (RESMAT) [20] also aimed to strengthen emergency response capacity but face limited coverage.

Rivers State experiences persistent disparities in emergency services provision. Despite Ministry of Health initiatives, inequalities in response times, workforce availability, and infrastructure persist between rural and urban areas [21]. International evidence shows that EMS access barriers follow recognisable patterns across contexts. Corscadden et al. identified cost, availability, and geographic accessibility as universal barriers [22], while Takele et al. found that lack of emergency contact knowledge significantly reduced utilisation [23], findings relevant to Nigeria's comparable LMIC context. Nto et al. found 57.8 % EMS awareness among Abuja residents, yet 91.4 % had never used emergency numbers [18].

Research on Rivers State EMS remains limited. One study conducted in Rivers State assessed only emergency obstetric care [24], while studies to understand rural-urban inequality in access and utilisation of EMS were yet to be seen. This study explores experiences with emergency medical services and examines delivery challenges across urban and rural areas. Objectives included: identifying systemic barriers to EMS access; analysing cultural and socioeconomic influences on utilisation; and examining community engagement roles in addressing EMS gaps.

## Methodology

### Study design and setting

This community-based qualitative study explored stakeholder experiences with EMS in Rivers State using the Levesque healthcare access framework [25]. The study was conducted in Port Harcourt, Rivers State capital. Rivers State operates a three-tier health system with 342 functional primary health centres [26]. The terrain includes urban centres and riverine communities, creating logistical EMS challenges [27].

Urban areas were defined as Port Harcourt metropolis and contiguous local governments (Port Harcourt City, Obio/Akpor, Eleme LGAs) with higher population density and concentrated infrastructure. Rural areas encompassed remaining local governments, including riverine and upland communities with limited infrastructure and sparse health facilities [27]. Participants were recruited from both categories to capture diverse geographic experiences.

### Study population

The respondents were purposively selected to ensure diverse representation, including respondents from both rural and urban areas and to capture detailed insights into their lived experiences and perceptions of the emergency medical services in their communities. They included policymakers, health care providers, community leaders, representatives of the telecommunication sector, representatives of the general public and non-governmental organisations (NGOs). Recruitment of participants involved outreach to local community organisations, faith-based institutions, health care facilities and community leaders through invitation letters. Consenting respondents participated in the study.

A total of 20 participants were engaged in one focus group discussion (Supplementary Appendix 1). The group was intentionally heterogeneous to incorporate multiple stakeholder perspectives and facilitate cross-sector dialogue. The sample size was informed by focus group best practices, the need for broad stakeholder representation, and practical considerations such as stakeholder availability [28]. Although larger than conventional homogeneous focus groups, this size allowed adequate coverage of nine stakeholder categories while remaining manageable for facilitation.

Inclusion criteria comprised healthcare personnel with at least one year of emergency medicine experience, management-level staff overseeing EMS, influential community leaders, service providers linked to EMS and clients who had previously accessed emergency care. Recruitment and data collection spanned three weeks. The inclusion of diverse participant categories in a single heterogeneous focus group was methodologically deliberate to ensure perspectives from multiple points in the emergency response chain. This approach aligns with the Levesque framework's emphasis on the interaction between supply-side (health system) and demand-side (population) characteristics [25]. While homogeneous focus groups might have yielded deeper within-group perspectives, our heterogeneous design facilitated examination of inter-sectoral disconnects and complementary viewpoints essential for understanding systemic access barriers.

### Data collection

The Levesque framework was used to guide the development of a semi-structured focus group guide. Two trained moderators experienced in qualitative research co-facilitated the discussion: a lead moderator guided the conversation while a co-moderator managed time, observed group dynamics, and ensured all participants had opportunities to contribute. The moderators had no prior relationship with participants, ensuring neutrality. The semi-structured focus group guide was developed through an iterative process: the research team initially mapped the five Levesque framework dimensions (accessibility, acceptability, availability, affordability, appropriateness) onto the Rivers State EMS context based on literature review and team expertise; draft questions were then reviewed by two external qualitative research experts and refined for clarity, neutrality, and cultural appropriateness; the guide was pilot-tested with three healthcare workers not included in the study, leading to minor revisions. A single two-hour in-person focus group session was conducted at a neutral, accessible venue in Port Harcourt. While acknowledging that data saturation is ideally confirmed through iterative data collection, our analysis revealed recurring themes across diverse participant types within this session, suggesting adequate thematic coverage for this preliminary study. At the session start, ground rules were established, emphasising confidentiality: participants were asked not to share others' contributions outside the session and to respect diverse viewpoints. Participants were addressed using generic titles (Sir/Madam) during discussions to minimise identification risk, though complete anonymity may not be guaranteed in focus group settings. Participants were not previously acquainted as a group, though some within the same professional category had prior professional relationships. This was disclosed during informed consent. The discussions were audio-recorded with permission from participants. Also, field notes were taken to capture key themes, non-verbal cues, and contextual observations.

### Data analysis

Audio recordings were transcribed verbatim using TurboScribe with subsequent manual verification. Transcripts were reviewed for accuracy using member checking to ensure validity, then imported into ATLAS.ti software for analysis. We employed a hybrid deductive-inductive analytical approach, combining framework-guided and data-driven coding [29]. The Levesque framework provided an initial deductive coding structure, with a priori codes derived from each dimension. However, we remained open to inductive emergence of themes not anticipated by the framework. During first-cycle coding, two researchers independently applied both deductive framework codes and generated inductive codes from unexpected content. A comprehensive codebook (Appendix 2) was developed and refined iteratively. Second-cycle coding involved grouping codes into themes, with some themes aligning with Levesque dimensions and others emerging inductively, such as "corruption," "NGO integration," and "technology opportunities".

Intercoder reliability was tested by independently coding 30 % of transcripts, achieving Cohen's kappa of 0.82. Discrepancies were resolved through consensus discussion. While the Levesque framework shaped our overall results structure due to its conceptual fit with emergent themes, several sub-themes and all "opportunities" themes emerged inductively from participant narratives rather than being predetermined by the framework.

### Ethical considerations

Ethical approval was obtained from the Rivers State Ethical Review Board (RSHMB/RSHREC/2024/125). Before the focus group discussions, participants were informed about the study details, and informed consent was obtained. All participants remained anonymous and de-identified in analysis, reports, and publications. All audio recordings and transcripts were stored securely on a password-protected system and were only accessible to the research team. The research team comprised public health professionals and EMS practitioners with varying affiliations to Rivers State Ministry of Health and Emergency Response Africa. This insider-outsider positioning offered contextual knowledge while requiring vigilance against assumptions. Team members reflexively acknowledged their professional stakes in EMS improvement, using peer debriefing to challenge interpretive biases and maintain analytical rigour [30].

## Results

The study examined barriers and opportunities in emergency medical services in both rural and urban areas using Levesque’s five-dimensional framework, selected for incorporating both population and health system perspectives:

### Accessibility

Participants highlighted delayed response times for emergency assistance, particularly in rural areas, with reports of prolonged waits. As one participant noted,” The ambulance took over two hours to arrive; by then, it was too late “P18 (rural resident), while another added, “Distances are too great for any effective EMS response.” P20 (emergency worker). Communication challenges further contributed to delays, including unreliable networks and inaccessible emergency lines. A telecommunication staff member explained, “Network issues often prevent timely calls for help.” P12 (rural resident and Telco staff), while another participant said, “Emergency lines are often unavailable when needed most.” P13 (urban resident and religious leader). Geographic barriers also restricted access due to poor road networks, especially during rainy seasons, as explained by a participant, “Villages are cut off during rainy seasons; no ambulance can reach us” P19 (community leader).

### Acceptability

The impact of religious beliefs was a recurrent theme. Some participants preferred faith-based interventions as opposed to medical interventions, as stated by a respondent, “Faith healing is seen as more powerful than medical help”, P5 (nursing officer), while another said, “My pastor told me to pray instead of going to the hospital”, P18 (rural resident). These preferences may be due to some misconceptions and myths about medical services that have become entrenched. One participant opined that, “Many believe ambulances are only for carrying dead bodies”, P9 (paramedic), while another said, “There’s this belief that hospitals will just take your money and do nothing”, P5 (nursing officer).

### Availability

Participants identified significant gaps in infrastructure supporting emergency care, particularly in rural settings. One participant stated, “Our clinic doesn’t even have stretchers, let alone an ambulance” P4 (rural nurse), while another noted that “Rural areas lack even the basic facilities for emergencies” P3 (health officer). A critical shortage of trained personnel further constrained service delivery. As a health personnel explained, “There are no qualified paramedics as per accreditation; even nurses are scarce” P8 (health trainer). These deficits collectively undermine the effective functioning of emergency medical services.

### Affordability

Financial barriers were a major deterrent to EMS use, with participants noting that high costs prevented timely care. As one respondent remarked, “How can you call an ambulance when you can’t afford the service?” P18 (rural resident), while another participant added, “People die because they can’t pay for care” P15 (healthcare worker). Participants also reported corruption and mismanagement, including inappropriate use of ambulances, as stated by a staff member, “Ambulances are used for personal errands by officials” P5 (nursing officer). Alongside chronic underfunding of EMS, as stated by a participant, “The government isn’t funding EMS enough; we’re left to fend for ourselves” P20 (emergency worker), the practice of requiring upfront payment also exacerbated delays in providing emergency care.

### Appropriateness

Participants also reported gender-based disparities in emergency care, with perceptions that women receive less priority during emergencies. One participant noted that, “Women’s health issues are often overlooked” P6 (urban nursing officer), while another added, “Men are prioritised over women during emergencies” P19 (community leader).

### Opportunities

While insights from the focus group discussions revealed challenges in EMS provision and utilisation, there were some opportunities identified to strengthen these services in the State. Training and capacity building emerged as a strategy, particularly through the engagement of community members as first responders to improve service provision and bridge the gap. As one participant noted, “We trained local volunteers to act as first responders” P1 (trainer), while another participant emphasised that “when people are trained, they can save lives before the ambulance arrives” P14 (public servant).

Public awareness and education were also mentioned. It was agreed that greater public awareness about the need for EMS services may improve service utilisation as a respondent stated, “There’s a need to educate communities about emergency response” P18 (rural resident). The application of technology was further identified as an important opportunity to enhance EMS efficiency. A participant stated that, “GPS tracking would improve ambulance efficiency”, P10 (telecommunication staff), particularly in remote settings. Finally, the integration of NGOs into EMS delivery was considered beneficial. NGOs were reported to enhance resources, with one staff member noting that “NGOs provide training and basic medical kits to our community” P2 (nursing service staff) and another stating that, “Without NGO support, rural EMS would collapse” P20 (emergency worker).

## Discussion

### Accessibility

Low public awareness of EMS emerged as a major challenge, spanning knowledge gaps about emergency medicine scope, contact procedures, and geographic barriers. This contrasts with Goto et al.'s Japanese study finding selective associations between health literacy and utilisation, where preventive services showed significant associations while emergency visits did not [31]. Contextual differences may explain this discrepancy; Japan's robust health system may enable emergency utilisation regardless of literacy level. However, Takele et al.'s [23] Ethiopian study found utilisation 3.6 times higher among individuals knowing emergency numbers. The difference between general health literacy effects (Goto) and EMS-specific knowledge effects (Ethiopia and Rivers State) underscores the importance of context-specific health education strategies.

Cultural beliefs and myths about emergency services played a role in EMS underutilization. Some participants mentioned that ambulances were sometimes viewed as vehicles for transporting dead individuals rather than resources for life-saving interventions. Previous studies have also highlighted how religious beliefs affected the use of healthcare services, even life-saving ones [32]. The study conducted across Nigeria used secondary data from the Nigeria Demographic Health Survey and identified that a woman's religious belief was statistically associated with utilisation of health services, including antenatal services [32]. During discussions, some individuals preferred seeking faith-based religious intervention as their first option rather than medical care, as the influence of cultural beliefs emerged powerfully in participant narratives. These factors indicate a need to incorporate culturally sensitive interventions and work collaboratively with religious and traditional leaders for orientation shifts.

Results confirmed that inadequate availability of EMS infrastructure was a key deterrent to utilisation, affecting both urban and rural areas, although the gravity was greater rurally. Most participants mentioned poorly equipped healthcare facilities, insufficient health personnel, and unavailable ambulances as affecting their utilisation of emergency care. A previous study highlighted that the quality of health clinics impacted the utilisation of health services and patient outcomes [33], although not specific to emergency medicine. Investments in training emergency staff and providing adequate equipment would likely improve provision and demand for emergency services and subsequent health outcomes.

Out-of-pocket expenditure associated with accessing healthcare services remains a major barrier to healthcare utilisation. Many residents were reported to be unable to access healthcare due to cost. Participants highlighted the lack of government-subsidised EMS schemes and high ambulance service costs as significant challenges. This aligns with a previous study showing an over 20 % increase in utilisation of general healthcare services when the government introduced subsidised healthcare services [34]. Furthermore, marginalised communities appeared to be disproportionately affected by high medical service costs; consequently, strengthening government financial investment and insurance schemes may improve equitable access to healthcare, especially in rural areas.

As the fifth dimension assessed, the study revealed that some participants felt appropriate care was not provided for some members of society, especially women. These testimonies reveal how appropriateness barriers extend beyond resource availability to encompass discriminatory practices that compromise equitable emergency care delivery, particularly for vulnerable populations, including women. This implies a need for further specialised training for emergency responders for such cases.

The deployment of technology, especially in tracking services and call enhancement, may benefit emergency service provision in the State. NGOs should work collaboratively with the government in providing emergency services. In areas where they currently operate, respondents reported markedly improved service compared to areas where they are currently absent.

These findings underscore the need for EMS policy reforms in Rivers State. Improving government involvement and financing, integrating NGOs in service delivery across all areas, and decentralising EMS management may be beneficial steps in improving emergency service provision and utilisation. Leveraging technology such as mobile apps, real-time GPS tracking, and response coordination is an area the Ministry of Health may need to consider. It is also suggestive that a multi-sectoral approach may be needed in improving emergency healthcare in Rivers State, involving government agencies, NGOs, other private sector players, and community partnerships for optimal outcomes. Formulating and implementing policies to address these identified barriers has the potential to significantly improve emergency medical services in Rivers State.

## Limitations

This exploratory qualitative study with 20 Rivers State participants provides rich contextual insights with potential transferability to similar LMIC settings but lacks statistical generalizability. The heterogeneous focus group design enabled multi-stakeholder perspective examination, though limited within-group depth exploration. Social desirability bias, particularly among government-affiliated participants, and inherent focus group anonymity limitations were addressed through neutral venue selection, independent moderation, and confidentiality protocols. Our hybrid deductive-inductive approach balanced theoretical structure with emergent theme openness, supported by strong intercoder reliability (Cohen's kappa=0.82).

## Conclusion

This study provides the first systematic qualitative examination of EMS barriers in Rivers State using Levesque's healthcare access framework, revealing systemic challenges across accessibility, acceptability, availability, affordability, and appropriateness dimensions, with rural areas disproportionately affected. Addressing these barriers requires policy-driven multi-sectoral approaches, including strengthened government intervention, NGO integration, enhanced public awareness, technology deployment, and community first-responder training. These context-specific, actionable recommendations provide a foundation for stakeholders to advance equitable emergency healthcare delivery in Rivers State and similar LMIC settings through collaborative implementation and sustained commitment to EMS system strengthening.

## Dissemination of results

Study findings have been shared with Rivers State Ministry of Health, to inform EMS planning; Emergency Response Africa (ERA), for operational refinement, and plans are underway to present findings at national health policy meetings and community forums for further advocacy and engagement.

## CRediT authorship contribution statement

**Adaeze Oreh:** Writing – review & editing, Conceptualization, Resources, Funding acquisition. **Folake Owodunni:** Writing – review & editing, Project administration, Conceptualization, Resources, Funding acquisition. **Oluwaseun Adebayo Adewunmi:** Writing – review & editing, Visualization, Conceptualization, Methodology, Writing – original draft. **Ihuoma Opelia-Ezeh:** Writing – review & editing, Conceptualization, Methodology, Writing – original draft. **Olufemi Onasanya:** Methodology, Data curation, Data curation. **Sylvanus Ojum:** Methodology, Data curation, Data curation. **Dede Siyeofori:** Methodology, Data curation, Data curation. **Kinikanwo Green:** Methodology, Data curation, Data curation.

## Declaration of competing interest

The authors declare **no conflict of interest** related to this manuscript.

## Appendix Supplementary materials

Image, application 1

## Footnotes

Supplementary material associated with this article can be found, in the online version, at doi:10.1016/j.afjem.2026.100946.
