# Supplementary material for: Rural-urban disparities in Emergency Medical Services: a qualitative study of barriers and opportunities in Rivers State, Nigeria
Source: Afr J Emerg Med. 2026 Jan 28;16(1):100946. doi: 10.1016/j.afjem.2026.100946 (PMC12874577; doi:10.1016/j.afjem.2026.100946)
Supplement: Supplementary file 1 [file mmc1.docx]

**APPENDIX**

**1. PARTICIPANTS’ SOCIODEMOGRAPHIC INFORMATION:**

| **Participants** | **Role/Profession** |
| --- | --- |
| Participant 1 | Rivers State Ministry of Health (RSMOH) |
| Participant 2 | Rivers State Ministry of Health (RSMOH) |
| Participant 3 | Health, Safety, and Environment (HSE) Officer (Energy Company) |
| Participant 4 | Nursing Officer, Primary Healthcare (PHC) |
| Participant 5 | Nursing Officer, Primary Healthcare (PHC) |
| Participant 6 | Nursing Officer, Secondary Health Care |
| Participant 7 | Nursing officer, Secondary Healthcare (Private Hospital) |
| Participant 8 | Department of Paramedic Studies, School of Health Technology |
| Participant 9 | Paramedic |
| Participant 10 | Staff of Telco |
| Participant 11 | Staff of Telco |
| Participant 12 | Staff of Telco |
| Participant 13 | Religious Leader (Pastor, Christian Church) |
| Participant 14 | Former member of the State House of Assembly, Rivers State |
| Participant 15 | Rivers State University Teaching Hospital (RSUTH) |
| Participant 16 | Staff of NGO |
| Participant 17 | Rivers State Police Command |
| Participant 18 | Community Leader |
| Participant 19 | Community Leader |
| Participant 20 | Rivers State Emergency Medical Services and Ambulance System (REMSAS) staff |

**2. COMPREHENSIVE CODEBOOK AND FREQUENCY TABLE**

**2.1 CODEBOOK**

The codebook includes all 19 codes identified from the thematic analysis, with descriptions, usage guidelines, and examples.

| **S/N** | **Code** | **Description** | **When to Use** | **Examples** |
| --- | --- | --- | --- | --- |
| 1 | DEL | Delayed Response; Long wait times for ambulances | Instances of delays in EMS arrival | “The ambulance took over two hours.” |
| 2 | INF | Inadequate Infrastructure; Poorly equipped or unavailable facilities | Discussions of resource limitations | “We don’t have stretchers.” |
| 3 | CUL | Cultural Beliefs; Misconceptions affecting EMS usage | Descriptions of beliefs hindering EMS use | “Ambulances are for dead bodies.” |
| 4 | TRA | Training; Need for trained responders | Discussions on training community responders | “Local volunteers can save lives.” |
| 5 | FIN | Financial Issues; Costs preventing access to EMS | Situations where affordability is discussed | “People die because they can’t pay.” |
| 6 | GEO | Geographical Barriers; Difficulties reaching remote areas | Mentions of transport or isolation challenges | “Villages are cut off during rains.” |
| 7 | KNOW | Public Awareness; Lack of knowledge about EMS services | Instances of misinformation or ignorance | “People don’t know the number to call.” |
| 8 | POG | Policy and Governance; Issues with bureaucracy or policy failures | Discussions on accountability or inefficiency | “Policies exist but aren’t followed.” |
| 9 | TECH | Technolgy; The role of digital tools in EMS | Discussions on apps, GPS, or tracking | “We need real-time ambulance tracking.” |
| 10 | REL | Religious Beliefs; Faith-based influences on EMS usage | When religion affects decisions in emergencies | “My pastor told me to pray.” |
| 11 | VUPO | Vulnerable Populations; Barriers for marginalized groups | Accessibility issues for elderly or disabled | “Disabled people can’t use ambulances.” |
| 12 | MIMA | Corruption; Mismanagement or misuse of EMS resources | Mentions of bribes or unethical practices | “Funds for EMS are embezzled.” |
| 13 | COME | Community Engagement; Involvement of locals in EMS | Discussions on awareness and local efforts | “Community volunteers are crucial.” |
| 14 | POV | Poverty; Financial struggles preventing EMS access | Discussions on affordability and priorities | “Food is prioritized over healthcare.” |
| 15 | GEN | Gender Disparities; Inequities faced by women | Issues related to cultural norms or neglect | “Women are ignored in emergencies.” |
| 16 | MH | Mental Health; Lack of EMS services for mental health | When discussing crises involving mental health | “We need trained personnel for mental health crises.” |
| 17 | NGOS | Role of NGOs; Contributions from NGOs to EMS | Instances where NGOs assist in EMS delivery | “NGOs provide basic medical kits.” |
| 18 | DECEN | Decentralization; Shifting EMS resources closer to users | Discussions about improving rural EMS access | “Services need to move closer to people.” |
| 19 | ACCESS | Accessibility Issues; Challenges in reaching EMS services | Physical or systemic barriers to access | “Rural areas don’t have any EMS coverage.” |

**2.2 FREQUENCY TABLE**

The frequency table shows how often each code was referenced during the analysis.

| **S/N** | **Code** | **Description** | **Frequency** |
| --- | --- | --- | --- |
| 1 | DEL | Delayed Response | 45 |
| 2 | INF | Inadequate Infrastructure | 40 |
| 3 | CUL | Cultural Beliefs | 32 |
| 4 | TRA | Training | 22 |
| 5 | FIN | Financial Issues | 28 |
| 6 | GEO | Geographical Barriers | 30 |
| 7 | KNOW | Public Awareness | 25 |
| 8 | POG | Policy and Governance | 20 |
| 9 | TECH | Technology | 18 |
| 10 | REL | Religious Beliefs | 15 |
| 11 | VUPO | Vulnerable Populations | 12 |
| 12 | MIMA | Corruption | 14 |
| 13 | COME | Community Engagement | 19 |
| 14 | POV | Poverty | 26 |
| 15 | GEN | Gender Disparities | 10 |
| 16 | MH | Mental Health | 9 |
| 17 | NGOS | Role of NGOs | 11 |
| 18 | DECEN | Decentralization | 13 |
| 19 | ACCESS | Accessibility Issues | 16 |
